# Stabilisation of thoracolumbar vertebral fractures and luxations in cats with a polyaxial screw/rod system

**DOI:** 10.1111/jsap.13879

**Published:** 2025-05-04

**Authors:** M. N. Çetin, Y. S. Şirin

**Affiliations:** ^1^ Department of Surgery, Faculty of Veterinary Medicine Burdur Mehmet Akif Ersoy University Burdur Turkey

## Abstract

**Objectives:**

The purpose of this study was to determine clinical and neurological data on the stabilisation of thoracolumbar region fractures or luxations in cats with the polyaxial screw rod system.

**Materials and Methods:**

The study included 16 cats with thoracic and/or lumbar fractures, luxations and/or instability. Data recorded for each patient included history, cause of vertebral fractures and dislocations, concurrent injury, time to surgery, neurologic examination and neurologic grading preoperatively and postoperatively (postoperatively, week 1, week 3 and month 6), surgical treatment, preoperative x‐ray and postoperative computed tomography imaging findings.

**Results:**

The region with the most localized lesions was T3‐L3 in ten cases and L4‐L7 in six cases. Polyaxial screws were placed unilaterally in 13 cases and bilaterally in three cases. A total of 50 polyaxial screws were placed in all cases. Of these, 44 polyaxial screws were placed optimally, four polyaxial screws were breached (screw diameter breach <2 mm) and two screws were broken. Broken screws did not require any revisions. Although 11 of the cases showed improvement in terms of neurological grading, no improvement was observed in five cases. The clinical outcome was excellent in four cases, functional in six cases and poor in six cases.

**Clinical Significance:**

The polyaxial screw rod system described here is a potential option for stabilization of thoracolumbar vertebrae in cats.

## INTRODUCTION

Vertebral fractures and luxations are one of the main causes of neurological damage in small animals (Beer, Knell, et al., 2020; Jeffery, 2010), almost always causing pain and neurological deficits (Grasmueck & Steffen, 2004; Jeffery, 2010). Cats can experience vertebral fractures alone or in conjunction with luxations (Vallefuoco et al., 2014), frequently affecting the lumbar vertebrae (Beer, Knell, et al., 2020; Bruce et al., 2008). Vertebral fractures or luxations constitute 6% of all spinal cord diseases in cats (Bali et al., 2009). External factors such as traffic accidents, falls from height, bites and gunshot wounds are the most common causes of vertebral fractures and luxations in cats (Eminaga et al., 2011; Orgonikova et al., 2021; Özak et al., 2018).

Treatment of vertebral fractures and luxations includes both surgical and conservative management (Vallefuoco et al., 2014). One common way to treat fractured or misaligned bones in cats is with tension bands (Voss & Montavon, 2004). The other is stabilization of the vertebral corpus using polymethylmethacrylate (PMMA) and pin or PMMA and screw fixation (Beer, Knell, et al., 2020). The goal of surgical treatment is the reduction of the vertebral segments, spinal cord decompression and rigid stabilization of the spinal canal (Bruce et al., 2008; Walker et al., 2002). Polyaxial pedicle screws are placed using standard screw placement techniques and provide rigid stability similar to a locking plate system (Monck et al., 2019). Dogs and humans frequently use polyaxial pedicle screws for spinal fusion and fixation (Nelson et al., 2021). On (date, November 11, 2023), the Pubmed database was searched using the following keywords: cat or feline + polyaxial screw rod stabilization, pedicular implantation, vertebral fracture, vertebral luxation, thoracolumbar fracture or luxation. These searches did not find any reports on polyaxial screw rod stabilisation for thoracolumbar vertebral fractures or luxations in cats.

This study aimed to determine the clinical and neurological effects of the use of a polyaxial screw rod system in the treatment of thoracolumbar vertebral fractures and luxations in cats.

## MATERIALS AND METHODS

### Animals

This study was approved by the Local Ethics Committee of ‘Burdur Mehmet Akif Ersoy University, Faculty of Veterinary Medicine’ (Decision Number: 922). Cats with neurological deterioration and persistent severe pain or with thoracolumbar fractures, luxations and/or instability were included in the study. However, patients with no or minimal deficits, stable fractures (fractures in only one compartment) preserved deep pain sensation and no severe pain were excluded.

### Examination

Data on history, breed, gender, age (<1.5 years; young, 2.5 to 7 years; adult, >7 years; mature), weight, cause of injury, concurrent injuries and time to surgery (<48 hours; acute, >48 hours; chronic) were recorded for all cats included in the study. All cats underwent a general physical examination and orthopaedic examination to identify any spinal injuries and accompanying non‐neurological lesions. All cats included in the study were neurologically examined preoperatively, and the lesion was localised. Neurological examination was repeated immediately postoperatively (after normal responses were obtained), in the first week and the third week. The Modified Frankel Scale was used to rate each case neurologically (Griffin et al., 2009) (Table 1).

**Table 1:** Modified Frankel scale used for neurological grading

Modified Frankel scale	
Grade 0	Paraplegia with absent deep nociception
Grade 1	Paraplegia with absent superficial nociception
Grade 2	Paraplegia with intact nociception
Grade 3b	Non‐ambulatory paraparesis (inability to bear weight on the pelvic limbs without support)
Grade 3a	Non‐ambulatory paraparesis (ability to bear weight on the pelvic limbs without support)
Grade 4	Ambulatory paraparesis
Grade 5	Normal gait with paraspinal hyperesthesia

### Radiography

After physical and neurological examination, orthogonal lateral and ventrodorsal (horizontal x‐ray technique) radiographs of the region of interest were obtained. Patients were carefully manipulated, and any spinal flexion or extension was avoided to prevent further vertebral injury. Radiographic evaluation was classified according to the location of the lesions in the vertebral column (C1‐C5, C6‐Th2, Th3‐L3, L4‐L7, S1‐S3). Fracture and luxation types were evaluated using the modified spinal trauma classification described by Bali et al. (2009). Here, lesions were classified as hyperflexion injuries, hyperextension injuries, subluxations, luxations, fracture‐luxations, transverse fractures, bursts or wedge compression fractures. Intervertebral disc space was also defined as normal or abnormal (visible dislocation, increase or decrease in space), and displacement of the caudal vertebral segment was classified as ventral or dorsal. Endplate involvement (endplate fractures and endplate–physeal fractures) was also assessed. The degree of vertebral dislocation was also evaluated preoperatively and postoperatively. The degree of dislocation was assessed by calculating the degree of displacement of the vertebral canal, ranging from 0% to 100%. The ventral edge of the vertebral canal was defined as a straight line from the most dorsal point on the cranial vertebral endplate to the most dorsal point on the caudal vertebral endplate (Bali et al., 2009). In lateral radiography, vertebral fracture stability was evaluated according to the three‐compartment theory. Fractures in the processus spinosus, processus articularis and the laminae were considered dorsal compartment; fractures on the dorsal surface of the vertebral corpus were classified as middle compartment fractures, and the remaining fractures were considered fractures of the ventral compartment (Platt, 2016).

### Anaesthesia, analgesia and antibiotherapy

Intravenous propofol (Propofol® 1%, Fresenius, 4 mg/kg iv) was used for induction. After the patient was intubated with an endotracheal tube of appropriate size, anaesthesia was maintained with sevoflurane (Sevorane®, Abbott). Preoperatively, cefazolin (Iespor®, Ulagay, 22 mg/kg iv) and fentanyl (Talinat®, Vem Medicine, 0.01 mg/kg iv) were administered.

### Polyaxial screw rod instrument set

**FIG 1:**
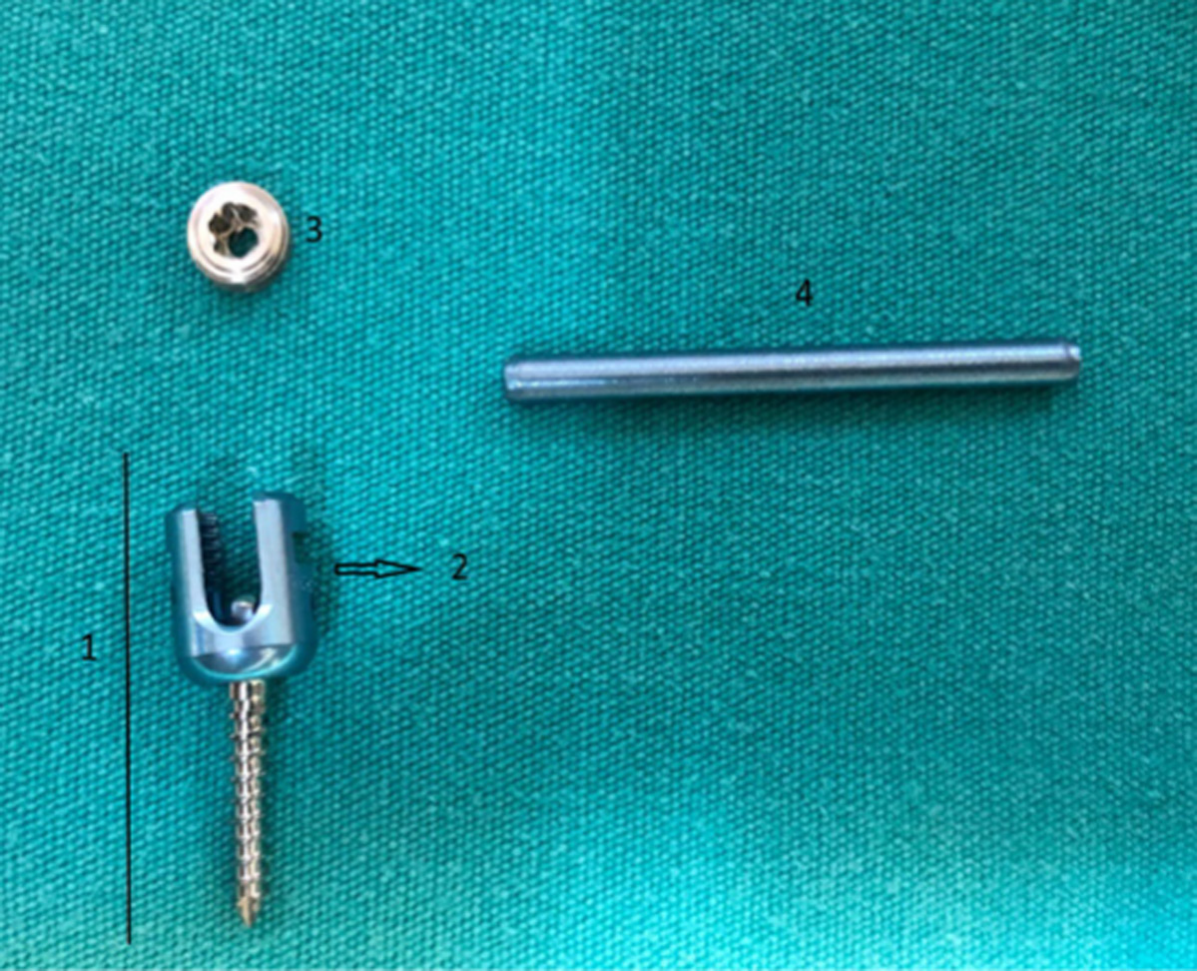
Polyaxial screw (1), tulip‐like head (2), set screw (3), connecting rod (4).

In all cases, stabilization was achieved using a polyaxial screw rod system (Travmavet, Vesta, Antalya, Turkey) for implantation. All cats used polyaxial screws with a diameter of 2 mm and varying lengths (12 and 14 mm). The head of the polyaxial screw has a tulip‐like shaft that allows 20° articulations. The polyaxial screw features a set screw that secures the rod to the screw. All cats used rods with a diameter of 2.5 mm, varying in length (30, 45 and 60 mm) (Fig 1).

### Surgical procedure

**FIG 2:**
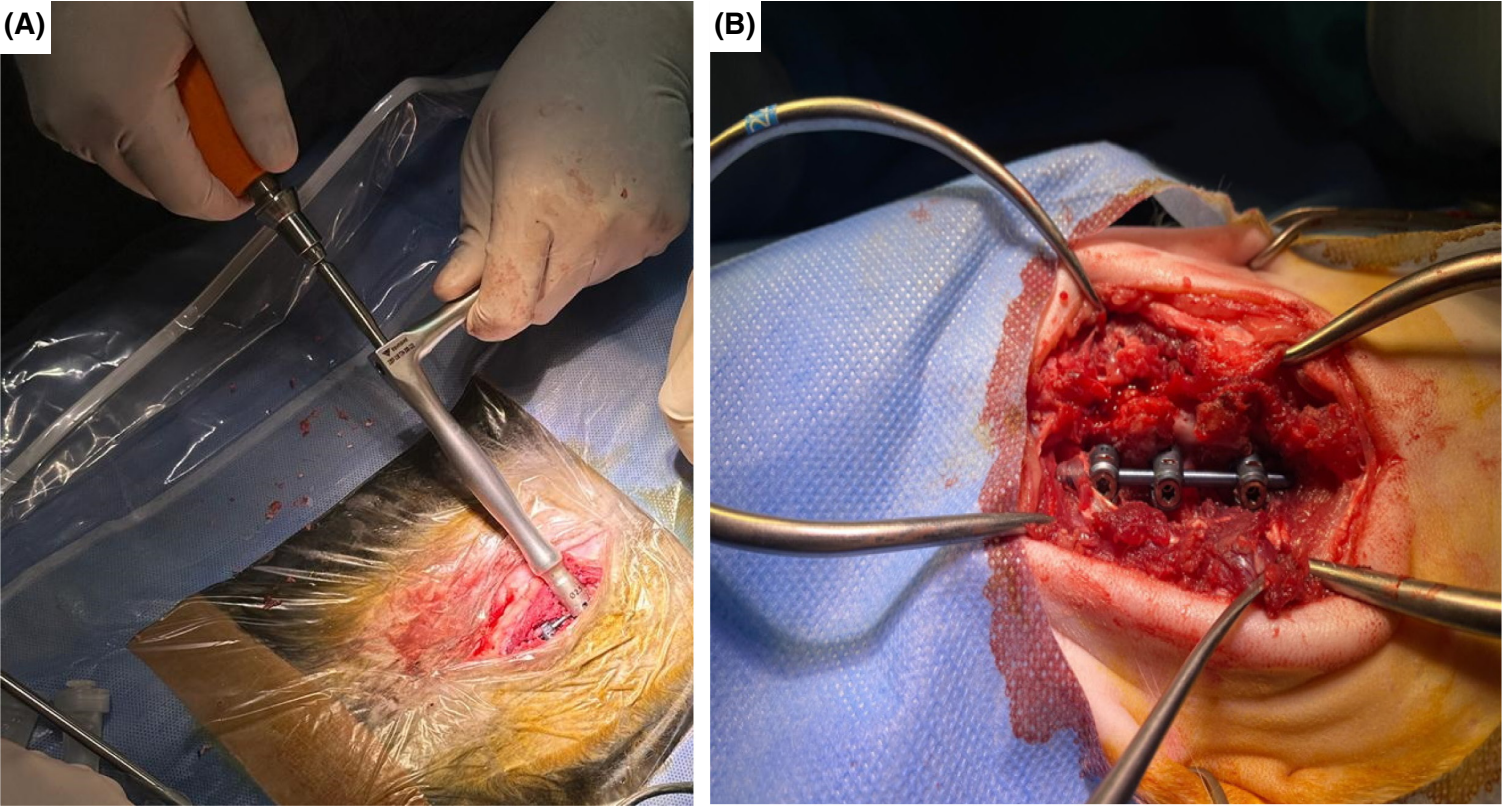
All set screws were tightened with a 1 Newton torque limiting device using the “L handle” (A), intraoperative view of stabilization with polyaxial screw and rod (B).

Following general anaesthesia, the cats were placed in sternal recumbency. The vertebrae were approached through a dorsal incision, exposing three vertebrae cranial and caudal to the lesion. The dorsal fascia was excised bilaterally on both sides of the four spinous processes centered over the lesion, and the supraspinous and interspinous ligaments between the spinous processes were preserved. Epaxial muscles were removed from the spinous processes, articular processes and pedicles. The transverse process was made visible. If decompression was necessary, the first decompression was performed by dorsal laminectomy. The vertebrae were reduced by distraction with small towel clamps applied to the spinous processes of the involved vertebrae. For L1‐L6 vertebrae, the level of the base of the transverse processes was determined as the polyaxial screw insertion points for the cranial, middle and caudal segments of the vertebrae, and the polyaxial screws were planned to be inserted approximately perpendicular to the spinous processes. For the T11‐T13 vertebrae, the insertion points were positioned at the level of the vertebrocostal joint, and the polyaxial screws were planned to be inserted approximately perpendicular to the spinous processes. The trajectory used for drilling was approximately perpendicular to the spinous processes to avoid damaging the vertebral canal dorsally and the vena azygos and aorta ventrally. At the T11‐T13 and L1‐L6 vertebral levels, a Kirschner wire (1.0 mm) was passed through the epaxial musculature to obtain the optimal trajectory for perpendicular placement of the polyaxial screw and was used to drill a pilot hole. At the T9‐T10 vertebral levels, the polyaxial screws were placed caudally to the vertebrocostal joint in the direction of the pedicle. At the level of the L7 vertebra, the ilium did not allow placement of the polyaxial screw at the level of the base of the transverse processes, so the polyaxial screw was positioned at the level of the pedicle. The screws were inserted into areas previously drilled with a Kirschner wire. After placing the screws, the connection was ensured by placing a rod, and tightening of all the set screws was achieved with a 1 Newton torque‐limiting device using a ‘L handle’ (Fig 2). After completion of stabilisation, deep muscles, superficial muscles, subcutaneous tissue and skin were routinely closed. After stabilisation, a soft bandage was applied to protect the incision line and cage rest was applied to each patient to support stabilisation.

### Computed tomography

**FIG 3:**
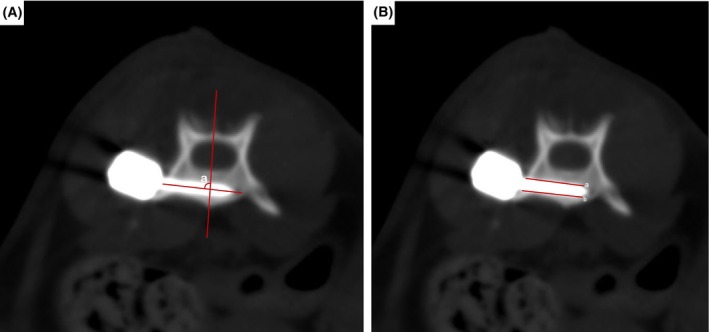
Demonstration of screw insertion angles (A), and bone stock a and b (B) measurements of polyaxial screws inserted into the vertebrae.

Postoperatively, sagittal, transverse and dorsal plane computed tomography (CT) images with a slice thickness of 2 mm were obtained for all cats using a computed tomography device (Toshiba Aquilion 64 Slice CT, Tokyo, Japan). These images were evaluated using the RadiAnt DICOM Viewer program. Screw insertion angle, bone stock of the screws and vertebral canal breach were evaluated by visualizing the long axis of each screw on CT images. The screw insertion angle was assessed as the angle between the sagittal plane and the line along the screw axis assessed on transverse CT images; the bone stock was assessed as the length of the medial (bone stock a) and lateral (bone stock b) screw surfaces covered by bone (Beer, Park, et al., 2020) (Fig 3).

The placement of the polyaxial screws in the corpus vertebrae was assessed using the Modified Zdichavsky Classification (I = optimally placed pedicle screw completely within the pedicle and vertebral corpus, IIa = partial penetration of the medial pedicle wall, IIb = full penetration of the medial pedicle wall entire screw diameter inside the vertebral canal), IIIa = partial penetration of the lateral pedicle wall and IIIb = full penetration of the lateral pedicle wall (whole of screw diameter outside the vertebral canal) (Elford et al., 2019).

### Postoperative management

Postoperatively, amoxicillin and clavulanic acid (Synulox®, Zoetis, 8.75 mg/kg sc) were administered once daily for 7 days and butorphanol (Butomidor®, Richter Pharma, 0.4 mg/kg sc) every 4 hours for at least 2 days or as long as the pain was present. A cage rest with a soft underlay was applied for 3 weeks. For those with neurogenic urinary dysfunction, the bladder was manually emptied 3 to 4 times a day. For 3 weeks, physical therapy, including heating, massage, passive range of motion, electrotherapy, cooling and balance exercises, was applied appropriately.

## RESULTS

### Signalement and examination findings

The breeds of the 16 cats included in the study were domestic short‐haired (*n* = 15) and Scottish Fold (*n* = 1). The mean age of the cats was 2.63 years (0.4 to 3 years), and the mean weight was 3.45 kg (1.5 to 5 kg). The study included cats who complained of falling from heights (*n* = 10), traffic accidents (*n* = 5) and dog bites (*n* = 1). Of these, ten were acute and six were chronic cases. The lesion was localized to T3‐L3 in ten cases and L4‐L7 in six cases. Only six cases (37.5%) recorded concurrent injury (Table 2).

**Table 2:** Signalement, examination and neurological findings

Case	Breed, gender, age, weight	Cause of injury	Duration	Fracture luxation location	Concurrent injures	Fracture and luxation type	Modified Frankel scale			
							Preoperative	Postoperative	First week	Third week
1	DSH, male, 9 m, 3 kg	RTA	Acute	TH3‐L3	–	Wedge compression	0	2	3b	5
2	DSH, male, 3 y, 2 kg	RTA	Acute	TH3‐L3	–	Luxation	0	0	0	0
3	DSH, female, 1 y, 2.5 kg	High‐rise fall	Acute	TH3‐L3	Pneumothorax	Fracture and luxation	0	0	0	0
4	DSH, male, 3 y, 3.5 kg	RTA	Acute	L4‐L7	Traumatic hip luxation	Subluxation	2	2	2	5
5	DSH, female, 2.5 y, 4 kg	High‐rise fall	Chronic	L4‐L7	–	Subluxation	2	2	4	5
6	DSH, female, 9 m, 3.4 kg	High‐rise fall	Acute	TH3‐L3	–	Wedge compression	2	2	3a	4
7	DSH, male, 6 m, 1.5 kg	RTA	Chronic	L4‐L7	Abdominal hernia	Subluxation	0	0	0	0
8	DSH, male, 6 m, 1.5 kg	RTA	Acute	L4‐L7	Os pubis fracture	Transverse	2	2	3b	3a
9	DSH, female, 2 y, 4 kg	Dog bite	Chronic	L4‐L7	Pneumothorax	Transverse	2	2	3b	3a
10	Scotish fold, female, 1 y, 3 kg	High‐rise fall	Chronic	L4‐L7	Cranial mandibular fracture	Transverse	0	0	0	0
11	DSH, male, 3 y, 4 kg	High‐rise fall	Acute	TH3‐L3	–	Wedge compression	0	1	2	2
12	DSH, female, 3 y, 4 kg	High‐rise fall	Acute	TH3‐L3	–	Subluxation	0	1	3b	3b
13	DSH, male, 9 m, 3 kg	High‐rise fall	Acute	TH3‐L3	–	Wedge compression	0	0	3b	4
14	DSH, male, 6 y, 4 kg	High‐rise fall	Chronic	TH3‐L3	–	Fracture and luxation	0	0	2	3b
15	DSH, male, 2 y, 3 kg	High‐rise fall	Chronic	TH3‐L3	–	Transverse	2	2	3b	4
16	DSH, female, 9 m, 3 kg	High‐rise fall	Acute	TH3‐L3	–	Luxation	0	0	0	0

DSH Domestic shorthaired cat, kg Kilogram, L Lumbar vertebrae, m Months, RTA Road traffic accident, TH Thoracic vertebrae, y Years, – Absent, + Available

### Neurological examination findings

According to spinal reflex examination, neurological localization revealed upper motor neuron findings in eight cats (50%) and lower motor neuron findings in eight cats (50%). In five cases that initially lacked a deep pain sensation, they regained it in the subsequent period. A modified Frankel scale evaluation showed improvement in 11 cases (68.75%) but not in five (31.35%) (Table 2). Urinary retention was present in 11 cases (68.75%), urinary incontinence was present in three cases (18.75%) and there was no problem with urinary dysfunction in two cases (12.5%). During the follow‐up period after discharge, there was no urinary dysfunction in ten cases (71%), urinary incontinence in two cases (14%) and urinary retention in two cases (14%).

### Radiographic findings

**FIG 4:**
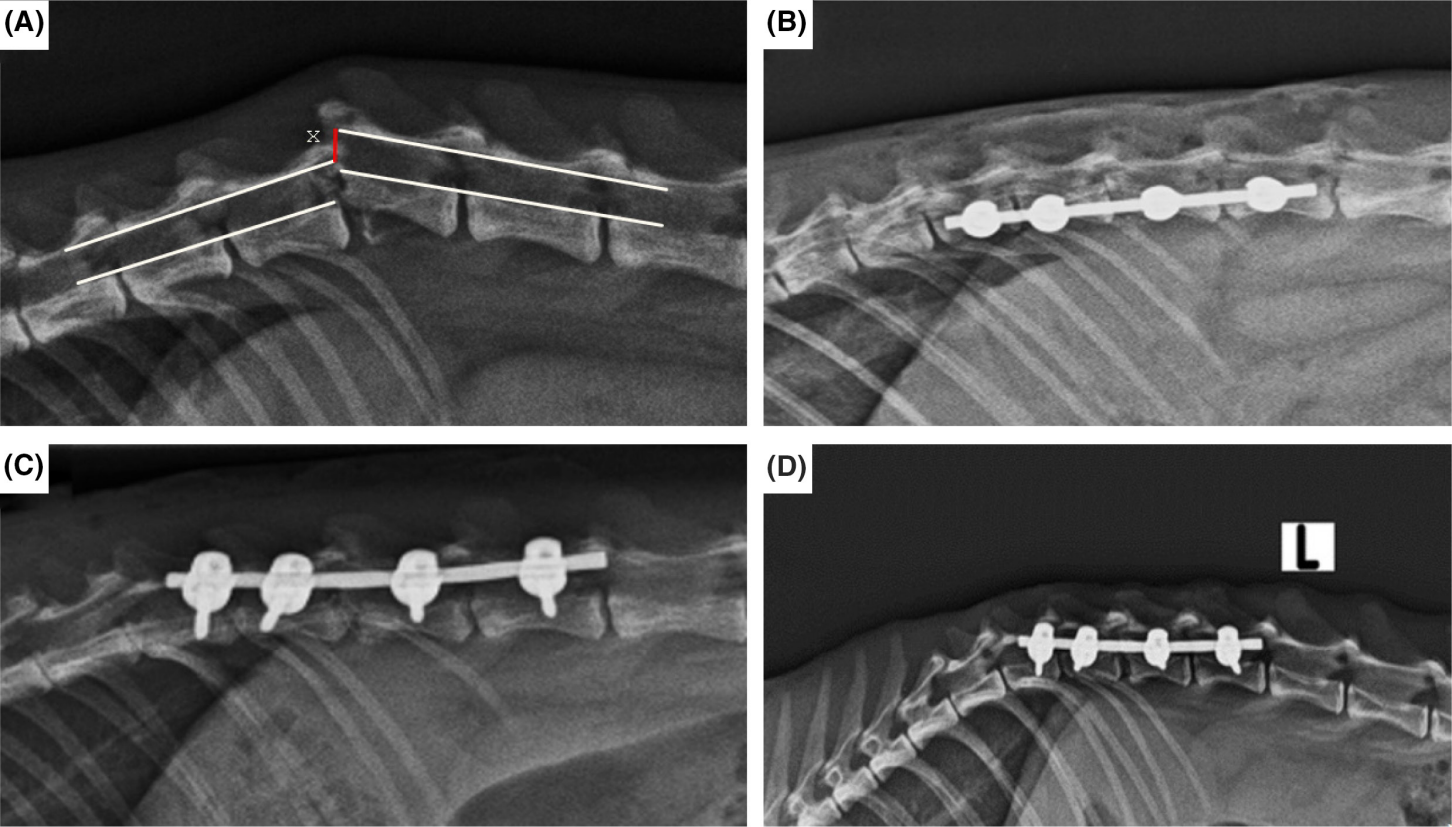
Radiographic evaluation of the degree of vertebral canal displacement (*x*) and vertebral alignment, preoperatively (A), postoperatively (B), postoperative first week (C), postoperative third week (D).

According to the Modified Spinal Trauma Scale, a wedge compression fracture was detected in four cases (25%), subluxation in four cases (25%), transverse fracture in four cases (25%), luxation in two cases (12.5%) and fracture and luxation in two cases (12.5%). The caudal segment of the vertebra was displaced dorsally in eight cases (50%) and ventrally in three cases (18.75%), while no displacement was detected in five cases (31.25%). In 11 cases (68.75%), the intervertebral disc distance decreased, in one case (6.25%), it increased and in three cases (18.75%), there was visible dislocation. When endplate involvement was evaluated, it was found that there was an endplate–physeal fracture in six cases (37.5%), and there was an endplate fracture in three cases (18.75%). In cases evaluated according to the theory of three compartments, three compartments were affected in 12 cases, ventral and middle compartments in one case, ventral and dorsal compartments in one case and only the ventral compartment in two cases. Preoperative assessment of vertebral canal displacement showed that the degree of dislocation ranged between 0% and 30% in eight cases and between 42% and 100% in eight cases. Postoperative evaluation showed complete resolution of vertebral canal dislocation in all cases and no disruption in vertebral alignment (Fig 4).

### Surgical findings

**FIG 5:**
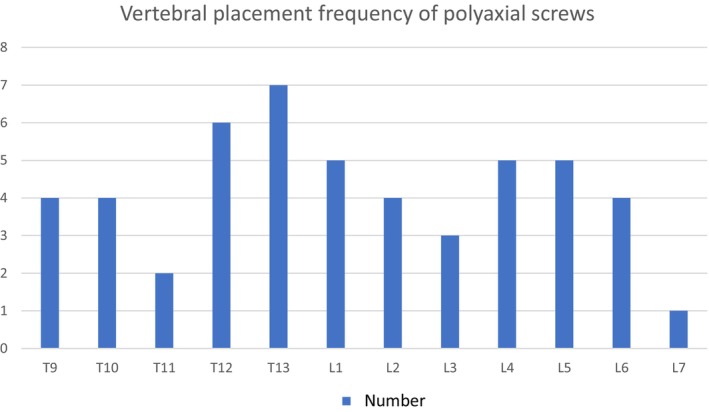
Graphical representation of vertebrae with polyaxial screws and number of polyaxial screws.

The mean duration until surgery was 81.9 hours (range: 12 to 240 hours). All cases were approached dorsally. Dorsal laminectomy was performed in ten cases. Polyaxial screws were placed unilaterally in 13 cases and bilaterally in three cases. Bilateral screws were placed in thoracic lesions in all cases. A total of 50 polyaxial screws were inserted (Fig 5). The diameters of all polyaxial screws and rods were 2 and 2.5 mm, respectively. Four cases used 12 mm‐long polyaxial screws, while 12 cases used 14 mm‐long polyaxial screws. Rods with a length of 30 mm were used in cases with two polyaxial screws, rods with a length of 45 mm were used in cases with three polyaxial screws, and rods with a length of 60 mm were used in cases with four polyaxial screws.

### Postoperative computed tomography

Polyaxial screws were placed bicortically in 12 cases and monocortically in four cases. Thirty‐seven polyaxial screws were placed bicortically and 13 polyaxial screws were placed monocortically.

The medial bone stock (a) and lateral (b) of the screws inserted into the vertebrae and the angles of screw insertion into the spine were determined. The mean bone stock a (9.8 mm) was found for the lumbar vertebrae, and the mean bone stock a (9.0 mm) was found for the thoracic vertebrae. The mean bone stock b (8.9 mm) was found for the lumbar vertebrae, and the mean bone stock b (7.5 mm) was found for the thoracic vertebrae. The mean screw insertion angle was 48.2° (9.7° to 89.2°) for the thoracic region and 67.4° (40.6° to 89.2°) for the lumbar region (Table 3).

**Table 3:** Representation of mean bone stock (a, b) and screw insertion angles of polyaxial screws inserted into vertebrae

Vertebrae	Average bone stock a (mm)	Average bone stock b (mm)	Average screw angle (°)
L1	10.0 (8.6 to 13.2)	7.5 (5.7 to 9.7)	70.6
L2	9.3 (8.2 to 10.4)	8.3 (7.4 to 9.4)	63.9
L3	10.2 (9.1 to 10.5)	10.3 (9.9 to 11.1)	83.1
L4	9.9 (7.2 to 11.9)	9.4 (4.9 to 13.0)	72.1
L5	12.3 (8.6 to 13.6)	10.7 (7.8 to 14.3)	71.2
L6	11.1 (9.8 to 12.3)	10.5 (9.4 to 12.5)	71.1
L7	5.8	5.6	40.0
T9	7.3 (6.1 to 8.9)	6.5 (4.9 to 7.6)	30.6
T10	9.0 (7.6 to 10.4)	8.9 (8.1 to 11.1)	13.9
T11	9.1 (7.9 to 10.3)	7.8 (6.7 to 8.9)	55.5
T12	9.8 (8.0 to 12.2)	6.2 (3.4 to 8.4)	67.9
T13	10.1 (8.1 to 12.5)	8.5 (7.2 to 10.5)	73.2

(mm) millimetre, (°) angle, minimum and maximum bone stocks of the screw are indicated in parentheses

**FIG 6:**
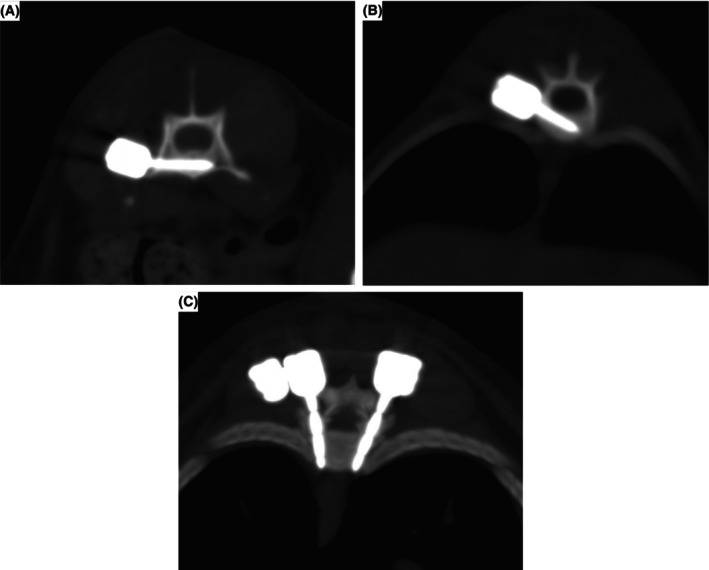
CT image of a polyaxial screw inserted at the level of the base of the processus transversus at L6 vertebra (grade I) (A), CT image of a polyaxial screw inserted at the level of the costal joint at T13 vertebra (grade I) (B), CT image of polyaxial screws inserted bilaterally into the pedicle at T10 vertebra (grade I) (C).

According to the Modified Zdichavsky Classification, most of the screws were placed optimally. A total of 23 polyaxial screws were placed in the thoracic region; 20 of these screws (87%) were classified as I, and the remaining three screws (13%) were classified as IIIA. A total of 25 polyaxial screws were placed in the lumbar region; 18 of these polyaxial screws (72%) were classified as I, four polyaxial screws (16%) as IIA (vertebral canal breach <2 mm) and three polyaxial screws (12%) as IIIA (Fig 6). Two polyaxial screws were not evaluated because they were broken. One of the broken screws was inserted in L6, and the other was inserted in L2.

### Postoperative results

The average hospital stay for all cases was 21 days. All cats were followed for an average of 6 months. In the postoperative follow‐up, the clinical outcome was ‘excellent’ in four cases (25%), ‘functional’ in six cases (37.5%) and ‘poor’ in six cases (37.5%). The poor outcome included four lumbar cases, one thoracic case and one thoracolumbar case. The functional outcome included two cases of thoracic and four cases of lumbar lesions. The excellent outcomes included two lumbar cases, one thoracic case and one thoracolumbar case.

## DISCUSSION

Polyaxial screw rod systems have been used in humans (Harms & Melcher, 2001), horses (Aldrich et al., 2018; Nelson et al., 2021), dogs (Marinho et al., 2018; Me'heust et al., 2000; Meij et al., 2007; Özak et al., 2018; Tellegen et al., 2015; Zindl et al., 2018), and sheep (Mutlu et al., 2011), but the literature review revealed that polyaxial screw rod systems have not been used to stabilize vertebral fractures and luxations in cats.

Since the pedicle screw rod system is designed for human vertebrae, the size of the screws is not suitable even for large‐breed dogs. The use of pedicle screws for adult humans has led to fractures of the lateral and medial walls of the pedicle. Pedicle screws for paediatric patients fit better into the adult canine vertebral pedicle (Meij et al., 2007). Paediatric pedicle screws with a diameter of 3.5 mm were successfully used in a dog with a thoracolumbar fracture, and it was determined that paediatric pedicle screw rod systems are suitable for large‐breed dogs (Özak et al., 2018). Cats can successfully use screws with a diameter of 2 mm, as evidenced by the perioperative achievement of rigid stability, the occurrence of a vertebral canal diameter breach in only four screws during the postoperative evaluation, and the absence of any vertebral alignment issues during the third postoperative week radiographic evaluation.

Thoracic and lumbar feline vertebrae are relatively small, and the use of safe corridors to ensure correct placement of implants such as screws and pins is crucial to prevent iatrogenic lesions to neuronal and vascular structures. Optimal corridors for safe implant placement, recently described in cats, provide the highest amount of bone uptake for bicortical implants on the one hand and increased surgical safety on the other (Vallefuoco et al., 2013). These corridors are located within the vertebral bodies. In the thoracic (T) vertebrae, the mean optimal angle from the midsagittal plane is 90.2°, with a maximum deviation angle of 10° dorsally and 8.8° ventrally from the insertion point. In the lumbar (L) vertebrae, the mean optimal angle from the midsagittal plane is 90.5°, with a maximum deviation angle of 8.4° dorsally and 7.6° ventrally from the insertion point (Vallefuoco et al., 2014). At the T9 and T10 vertebral levels, the pedicles were preferred as a safe corridor for the screws due to the ease of application and the observation that adequate stability was achieved perioperatively when the screws were placed bilaterally. The radiographic evaluation during the third postoperative week showed no deterioration in vertebral alignment. At this level, the placement of the screws in the pedicle resulted in narrower angles, and the subsequent period's lack of vertebral alignment issues demonstrated that the bilaterally placed screws had sufficient bone stock. Simultaneously, the CT images revealed no vertebral canal breach at these vertebral levels. At the T11‐T13 and L1‐L6 vertebral levels, the screws were planned to be inserted approximately perpendicular to the vertebral corpus, but this was not always achieved. Four screws at the lumbar vertebral level showed exceedingly small vertebral canal breaches in the postoperative CT images. It was observed that the screws could be placed at narrower angles, and this did not cause any problems. A subsequent radiographic evaluation confirmed the placement of the screws at these vertebral levels with sufficient bone stock, without any distortion of the vertebral alignment. In the L7 vertebra, the screw could only be placed at a steeper angle due to the ilium, resulting in a narrower angle and less bone stock compared to other lumbar regions. However, radiographic evaluation at 3 weeks postoperatively showed no deterioration in vertebral alignment (Table 3).

In humans, the clinical relevance of screw perforation remains controversial, as does the requirement for surgical revision when a breach is detected without concurrent neurological deficits. In man, screws perforating the medial pedicle wall of over 4 mm are considered high‐risk for damage to neurovascular structures in humans; perforations of 2 to 4 mm are considered lower‐risk; and perforations of <2 mm are considered to be within the ‘safe zone.’ However, no such clinical guidelines are specifically available for dogs. Because the canine pedicle is smaller than that of humans, screw breaches of <4 mm may already impair neurological function (Beer, Park, et al., 2020). Based on the modified Zdichavsky classification, the pedicle screw was thought to have penetrated the vertebral canal if it went more than 50% of the way through from the side to the middle. If more than 50% of the screw diameter was inside the pedicle and more than 50% of the screw diameter was outside the vertebral corpus, it was considered excessive deviation (Gougeon & Meheust, 2021). In this study, only four screws (8.3%) had <2 mm of vertebral canal breaches. In each of the three vertebral canal breach cases, deep pain sensation was initially absent. In one case, a deep pain sensation returned, but in the other two, it did not. Therefore, it was impossible to determine if a screw breach or pre‐existing spinal cord damage prevented the cats from regaining deep pain sensation. Accordingly, it was thought that it would be more accurate to evaluate whether a vertebral canal breach of <2 mm in cats would impair neurological function in more cases, and no interpretation could be made based on the data obtained. However, given that cats' vertebral canal diameter is smaller than that of humans and dogs, it is important to consider the potential danger of smaller screw diameters. Moreover, according to the Modified Zdichavsky classification, vertebral canal breaches were considered insignificant, considering that no screw diameter penetrated more than 50% of the vertebral canal.

A study investigating cervical stabilisation with polyaxial screws in horses suspected implant failure in one horse due to insufficient tightening of the fixation screw. The fixing screw is responsible for locking the entire structure (Aldrich et al., 2018). In the study, after all the fixing screws were placed, the final tightening process was performed by applying a torque force of 1 Newton to all the fixing screws. Therefore, the study encountered no such problems.

The results of load‐failure tests of polyaxial pedicle screws in humans show that the weakest point of the structure is the screw head connection (Fogel et al., 2003). In a study, it was thought that some of the screw fractures were due to the small bone stock (Beer, Park, et al., 2020). Implant failure (broken screw) in horses was incidentally detected in two cases but did not require treatment or euthanasia as described following implant fracture by other methods used to stabilize the cervical vertebrae (Pezzanite et al., 2021). In this study, both screws broke at the screw head attachment point, reinforcing the idea that the screw head attachment is the weakest point as determined by the results of load‐failure tests in humans. The fractured screw placed in L6 was thought to have fractured due to the small bone stock (bone stock a: 7.47 mm, bone stock b: 1.5 mm). However, the fractured screw in L2 retained a sufficient amount of bone stock (bone stock a: 12 mm, bone stock b: 9.5 mm), suggesting that the fracture may have resulted from a biomechanical load. The broken screw did not require any extra treatment or euthanasia, and there was no change in the position of the screws or vertebrae.

This study has limitations that should be highlighted. The lack of preoperative advanced imaging in the study limited our knowledge of spinal cord injury. Although the lack of myelography may be seen as a limitation, myelography was not used because of the need to manipulate the vertebrae during contrast injection (Kinns et al., 2006), possible side effects including increased pressure in the spinal canal and seizures (McKee, 2016; Olby, 2012), and the lack of data to detect compressive extradural lesions of the spinal cord in cats compared to dogs (Eminaga et al., 2011). Another limitation was that information about the animals during the six‐month follow‐up period was obtained by calling the owners, and radiographs or neurological examinations could not be performed during six months. Postoperative clinical results were evaluated according to feedback from the owners.

This is the first case series to radiographically and tomographically evaluate the feasibility of the polyaxial screw rod system for the stabilisation of thoracolumbar vertebral fractures in cats. This study demonstrated the applicability of the polyaxial screw rod system for thoracolumbar vertebral stabilisation, as well as the system's satisfactory stability that promotes healing. This surgical stabilisation method offers a promising alternative to other surgical stabilisation methods.

## Author contributions

**M. N. Çetin:** Conceptualization (equal); data curation (lead); formal analysis (lead); investigation (lead); methodology (equal); project administration (equal). **Y. S. Şirin:** Conceptualization (equal); data curation (supporting); formal analysis (supporting); investigation (supporting); methodology (equal); project administration (equal).

## Conflict of interest

None of the authors of this article has a financial or personal relationship with other people or organisations that could inappropriately influence or bias the content of the paper.

## Data Availability

The datasets used and/or analyzed in the current study are available from the corresponding author upon reasonable request.
